# Co-operative intermolecular kinetics of 2-oxoglutarate dependent dioxygenases may be essential for system-level regulation of plant cell physiology

**DOI:** 10.3389/fpls.2015.00489

**Published:** 2015-07-15

**Authors:** Siddhartha Kundu

**Affiliations:** School of Computational and Integrative Sciences, Jawaharlal Nehru UniversityNew Delhi, India

**Keywords:** 2-oxoglutarate, facial triad, co-operative kinetics, system-level, iron deficiency, non-linear regression

## Abstract

Can the stimulus-driven synergistic association of 2-oxoglutarate dependent dioxygenases be influenced by the kinetic parameters of binding and catalysis?In this manuscript, I posit that these indices are necessary and specific for a particular stimulus, and are key determinants of a dynamic clustering that may function to mitigate the effects of this trigger. The protein(s)/sequence(s) that comprise this group are representative of all major kingdoms of life, and catalyze a generic hydroxylation, which is, in most cases accompanied by a specialized conversion of the substrate molecule. Iron is an essential co-factor for this transformation and the response to waning levels is systemic, and mandates the simultaneous participation of molecular sensors, transporters, and signal transducers. Here, I present a proof-of-concept model, that an evolving molecular network of 2OG-dependent enzymes can maintain iron homeostasis in the cytosol of root hair cells of members of the family Gramineae by actuating a non-reductive compensatory chelation by the phytosiderophores. Regression models of empirically available kinetic data (iron and alpha-ketoglutarate) were formulated, analyzed, and compared. The results, when viewed in context of the superfamily responding as a unit, suggest that members can indeed, work together to accomplish system-level function. This is achieved by the establishment of transient metabolic conduits, wherein the flux is dictated by kinetic compatibility of the participating enzymes. The approach adopted, i.e., predictive mathematical modeling, is integral to the hypothesis-driven acquisition of experimental data points and, in association with suitable visualization aids may be utilized for exploring complex plant biochemical systems.

## Introduction

**Figure 1:**
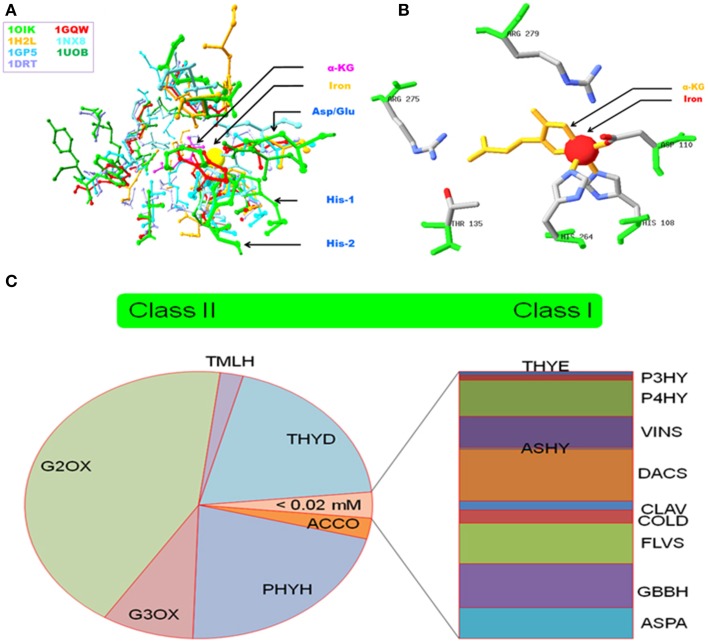
**Salient features of 2OG-dependent non-haem Fe (II) dioxygenases. (A)** Superposition of active site amino acids of select enzymes **(B)** Experimentally validated residues of Alkylsulfatase B that participate in iron coordination, 2OG binding, and/or substrate interaction, and **(C)** Categorization of enzymes based on thresholds, i.e., *Km*_Fe_,-values mined from literature. Abbreviations: PDBIDs^*^: 1OIK, Alkylsulfatase B (Muller et al., 2004); 1GQW, Taurine dioxygenase (Elkins et al., 2002); 1H2L, (Elkins et al., 2003); 1GP5, Anthocyanidin synthase (Wilmouth et al., 2002); 1UOB, Deacetoxycephalosporin synthase (Valegard et al., 2004); 1DRT, Clavaminate synthase 1 (Zhang et al., 2000); 1NX8, Carbapenem C (Clifton et al., 2003). Nomenclature^#^ (This work): THYE, Thymine dioxygenase; P3HY, Prolyl 3-hydroxylase; P4HY, Prolyl 4-hydroxylase; VINS, Deacetoxyvindoline 4-hydroxylase; ASHY, Asparagine hydroxylase; DACS, Deacetoxycephalosprorine synthase; CLAV, Clavaminate synthase I; COLD, Procollagen lysine 5-dioxygenase; FLVS, Flavonol synthase; GBBH, gamma-butyrobetaine dioxygenase; ASPA, Aspartyl-asparaginyl hydroxylase; ACCO, Aminocyclopropanecarboxylate oxidase; PHYH, Phytanoyl CoA hydroxylase; G3OX, Gibberellin 3beta-dioxygenase; G2OX, Gibberellin 2beta-dioxygenase; TMLH, Trimethyllysine hyroxylase; THYD, Pyrimidine-deoxynucleoside 2-dioxygenase.

The alpha-ketoglutarate (AKG) dependent non-heme Fe (II) dioxygenase superfamily is characterized by variable reaction chemistry and exceptional substrate versatility. This has been attributed to: a relaxed co-ordination geometry for ferrous iron, the formation of an exceptionally reactive, transient ferryl species [Fe(IV)=O], and several sequence specific features (Price et al., 2003a,b; Hausinger, 2004; Clifton et al., 2006; Kundu, 2012). The hexadentate interaction with Fe (II) includes the residues His-X-Asp/Glu-Xn-His (facial triad), 2OG, and a displaceable water molecule in a 3:2:1 association (Figures 1A,B). In contrast to the conserved binding profile of these enzymes for iron, the active site amino acids for 2-oxoglutarate and their cognate substrate(s) are distinct and protein specific. Plant AKG-dependent enzymes, participate in flavonoid and alkaloid biosynthesis (catalytic and regulatory), maintain cell architecture (direct, prolyl hydroxylases; indirect, 2S-flavonols), and influence seed dormancy (Kawai et al., 2014). Recent laboratory data also suggests that the o-hydroxylation of feruloyl-CoA (Feruloyl-CoA 6′-Hydroxylase1; EC 1.14.11.) results in compounds that are able to directly facilitate ferric iron absorption in alkaline soils as part of strategy II, i.e., non-reductive assimilation (Kobayashi and Nishizawa, 2012; Schmid et al., 2014).

The presence of a shared double-strand-beta-helical (DSBH) fold, notwithstanding, the members of the 2OG-dependent superfamily of dioxygenases have little in common with regards to the substrates converted and the reaction chemistries deployed. The biochemical significance of this divergence has been highlighted earlier, with the development of a classification schema based on functional homology, and the description of a combinatorial network of AKG-dependent enzymes to accomplish system-level function (Kundu, 2012, 2015). In the former, profile hidden markov models (pHMMs) of enzymes with a similar substrate(s) and/or reaction mechanism were constructed, and utilized to create a repository (DB2OG) of probable 2OG-dependent sequences (Kundu, 2012, 2015). Thus, SULF (*N*_SULF_ = 3), a pseudo-family comprising taurine dioxygenase, alkylsulfatase K, and the YSD proteins, are characterized by an additional active site His residue that interacts with a sulfur atom present within the structure of their cognate substrates. Similarly, DSAT (*N*_DSAT_ = 6), whose members, *viz*., the carbapenem- and clavaminate-synthases and select enzymes of the 2S-flavonol biosynthetic pathways, can facilitate the simultaneous removal of two protons, thereby, inserting a double bond between adjacent carbon atoms. A recently concluded study emphasized the importance of dispersed sub-cellular locales, differential domain distribution, and a variegated substrate profile, in the genesis of transient and task-driven aggregates of α-ketoglutarate-dependent enzymes (Kundu, 2015). These results, in a major departure from the one-protein-one-function norm, were established using a combination of mathematical and computational ideas. Thus, while prolyl 4-hydroxylase and asparaginyl hydroxylase can actuate a downstream response to hypoxia, the gibberellic oxidases are responsible for the balance between the high- and low-activity GAs (Hedden and Phillips, 2000a,b; Hewitson et al., 2002). However, despite these insights, the pattern and determinants of this synchronized activity remain speculative. The cardinal premise of this work is that an arbitrary, exogenous stimulus can dictate the dynamics of complex molecular networks, and that this stimulus-driven co-operation amongst 2OG-dependent dioxygenases is critical to its role as a systems-player. The exposition, *vide infra*, discusses the development and validation by kinetic parameterization, of an event that could link a perturbation, such as the unavailability of a critical micronutrient, with a stable biochemical modifier of AKG-dependent enzymatic activity. The requirement of active site Fe(II) for this superfamily is invariant, is dependent on the labile iron pool, and can serve as a contributory influence to this real-time clustering.

Iron is an important trace element and in small quantities (<100 ppm), is needed for the proper functioning of several biologically relevant proteins. Although, an oversimplification, the bio-organic-metal interactions for iron maybe categorized as haem- or non-haem based. Deficiency states, which reflect an extinct or unusable labile iron pool (LIP), are referred to as iron/magnesium-dependent chlorosis, with progressive yellow/white discoloration. The LIP, is numerically expressed as the sum of chelated- and free-iron forms, and has traditionally been difficult to estimate directly. Absorption of iron from soil is dependent on the pH, with recovery rates proportional to the fraction of ferrous iron prevalent. This would translate into maximal assimilation if, the hydrogen-ion concentration of the surrounding rhizosphere were in the acidic range (pH 5.0–6.5 units). The apparatus to facilitate membrane iron transport, at least in plants, appears to have evolved as mutually exclusive events. Strategy I-absorption (reductive), is seen in non-gramineae, whilst, the alternate mechanism (strategy-II) is common in cereal members of the gramineae. This delineation, is however, pedantic with several species exhibiting a dual propensity for either. Thus, non-graminaceous crops are able to reduce iron externally, as well as effect a limited mobilization of Fe(III) in high pH soils using coumarins (Rodriguez-Celma et al., 2013; Fourcroy et al., 2014; Schmid et al., 2014). Similarly, poaceae cereals utilize phytosiderophores in a calcareous environment to assimilate ferric chelates, but are competent to implement strategy-I under specialized conditions (flooded paddy fields; Ishimaru et al., 2006; Kobayashi and Nishizawa, 2012).

Predictive mathematical models (static and simulations), may be regarded as abstract representations and may afford mechanistic perspectives into the behavior of complex biochemical systems, of which the AKG-dependent superfamily is a classic exemplar. The initial step is the formulation of an equation with the dependent variable as a linear- or non-linear combination of single- (regression) or multiple- (generalized linear models, GLMs; artificial neural networks, ANNs) independent contributing variables. Coefficients are estimated separately using random numbers from known probability distributions (modified Monte Carlo methods) or training/testing datasets (supervised learning). Stochastic simulations provide an unbiased evolution of trajectories despite being analytically intractable. Here, the chemical master equation is solved using Gillespie's stochastic simulation and finite state projection algorithms, the methods of -sliding windows and -conditional moments, complex chemical Langevin equation, among several others (Munsky and Khammash, 2006; Gillespie, 2007; Wolf et al., 2010; Hasenauer et al., 2014; Schnoerr et al., 2014).

The reference cell chosen for this study was the root hair of the gramineae (cereals). These short-lived, epidermal extrusions arise from the zone of maturation, and in response to an exigent event, exhibit progressive loosening of the cell wall, elongation, exudation, and complex uptake mechanisms. Biochemically, too, the cell affords a stable cellular milieu, has several specialized 2OG-dependent enzymes, and possesses distinct macromolecules that can maintain iron homeostasis. In this monograph, I have analyzed available kinetic data (*Km_Fe_*, *Km_2OG_*) of experimentally validated members of the AKG superfamily, and formulated composite models of the same. The results, both, a posteriori, and computed, when interpreted within the defined framework of the root hair cell, and with pre-computed threshold values, clearly suggest that depressed availability of exogenous iron can result in harmonized cellular 2OG-dependent activity and contribute to the detection and initiation of appropriate counter- measures following a reduction in Fe(II) levels, disjointly, as well as in tandem. Additional findings indicate that reprisal of this novel role, is dependent on a scaffold of interdependent reaction pathways, active site properties of the participating enzymes, and the presence of key convergence nodes. The kinetic data (association, dissociation, and catalysis) utilized, constitute easily verifiable observations, and appears fundamental to the genesis of a dynamic network of select non-haem alpha-ketoglutarate dependent Fe (II) dioxygenases that can accomplish system-level function.

## Materials and methods

### Software and computational tools

Sequences and structural data were downloaded from the Uniprot and PDB (http://uniprot.org; http://www.rcsb.org) databases. Distribution of 2OG-dependent catalytic domains of select sequences were predicted using the server module of H2OGpred (http://comp-biol.theacms.in/H2OGpred.html; Kundu, 2012). Miscellaneous computational resources included BRENDA (a repository of biochemical data; Schomburg et al., 2002), the STRAP suite of programs (phylogenetic trees and alignment files; Gille and Frommel, 2001), and the SPDBV (superimposition and visualization of structural data; Guex and Peitsch, 1997). Data processing (parsing, sorting, computing, and formatting) was accomplished using PERL scripts developed in-house.

### Datasets and *in silico* experiments

Enzymes with available kinetic data, catalytic (*Km*) and binding association (*Ka*) values for iron and 2-oxoglutarate (Table T1 in Supplementary Material) were compiled and analyzed (*D*_Fe_, *N* = 17; *D*_AKG_, *N* = 29). Multiple values for an enzyme, with data from either different organisms or variable experimental conditions excluding mutagenesis studies, were averaged. Partitioning, for further computational experiments were in accordance with previously determined ferrous iron levels (Urzica et al., 2012; *T* ∈ {0.0005, 0.001, 0.003, 0.02}). Thresholds were also determined using the Monte Carlo method. Numbers were drawn randomly from the open interval (0, 1), i.e., 0<x<1, and scaled with ranges bounded by threshold values. Each experiment was done in triplicate and repeated 500 times. The quasi-datasets obtained were summarized with descriptive statistics. Quartile values of the empirically determined kinetic data of enzymes (*q*(*D_Fe_*)) and the predicted values for the model *Km_NLR_Fe_*, constituted the complete set of bounds on which inferences about 2OG-dependent behavior were based (*t* ∈ *T* ∪ *q(D_Fe_)* ∪ *Km_NLR_Fe__*).

### Mathematical models

Non-linear regression (NLR) is an established statistical method for predicting values of a dependent variable. Here, the coefficient of determination (*R*^2^) serves as a measure of model selection. There is a fair volume of publically available kinetic data for ferrous iron, 2OG, and O_2_ (Table T1 in Supplementary Material). Since, molecular dioxygen is an essential reactant and is incorporated into the substrate, the pertinent values were not considered further. Compiled kinetic data points for Fe (II) and 2-oxoglutarate were fitted to several equations and the closest approximate to unity was chosen for further analysis.

### Model evaluation and derived kinetics

Model evaluation was carried out using the chi-squared test (χ^2^). For these calculations the observed values were individual enzyme systems at a threshold value (*V*^0^_*enzyme*_ (*t*)), while the expected value was the behavior of the model at the same threshold (*V*^0^_*model*_(*t*)).

(1) $$\chi^{2}=\sum_{i=1}^{i=17}\left\{{\left(V_{i}^{0}(t)-V_{model}^{0}(t)\right)}^{2}/V_{model}^{0}(t)\right\}$$

Derived velocity maxima for the enzymes was formulated as a modification of the existing Michaelis-Menten (MM) equation:

(2) $$V_{enz}^{0}=\left(0.5\right)\left(t/Km_{enz}\right)$$

t : = threshold iron concentration

Km : = MM constant

enz ∈ {NLR_Fe_, individual enzymes}

## Results

### Classification schema

The kinetic data for ferrous iron was the basis for a categorization schema (Figure 1C). Class I (*N* = 11, high affinity, *Km* ≤ 0.02 *mM*) enzymes participate in proline hydroxylation, vinca alkaloid synthesis, and the flavonol biosynthetic pathways. Class II (*N* = 6, low affinity, *Km* > 0.02 *mM*) includes the ethylene-forming ACCO, and the gibberellic acid 3- and 2- β-dioxygenases. This data suggests that 2OG-dependent dioxygenases have differential affinities for iron, a factor that might translate into a graded response to fluctuating cytoplasmic levels.

### Curve fitting and model selection

**Figure 2:**
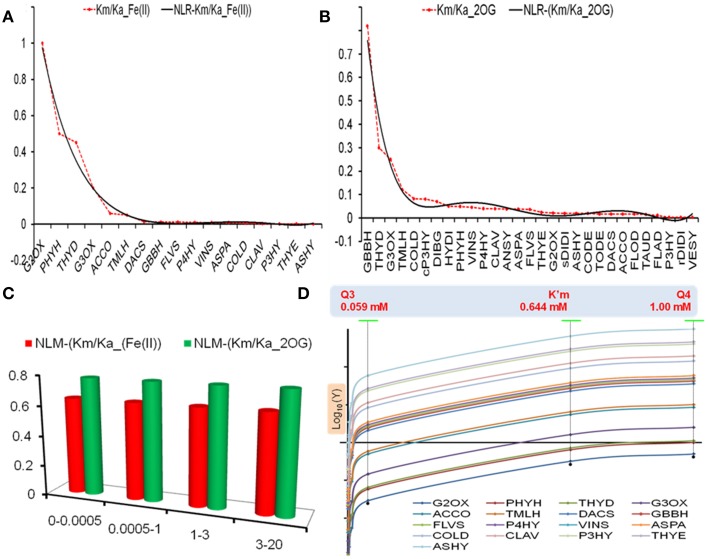
**Model selection and analysis. (A,B)** Non-linear regression models of *Km*_Fe_ and *Km*_AKG_ values in accordance with Equations (3) and (4) **(C)** Analysis of model robustness using a Monte Carlo approach, and **(D)** Predicted *V*^0^_*enzyme*_ values at different thresholds for select enzymes. Here the ordinate axis is a Log_10_ representation.

A non-linear regression curve for each dataset, i.e., *D*_Fe_ and *D*_AKG_, was fitted using a 6-degree polynomial (Figures 2A,B) as under.

(3) $$\begin{array}{l} {\text{Km}}_{\text{Fe}}=7E-07{\text{x}}^{6}-4E-05{\text{x}}^{5}+0.001{\text{x}}^{4}-0.016{\text{x}}^{3}\\ \;+0.141{\text{x}}^{2}-0.707\text{x}+1.556 \end{array}$$

(4) $$\begin{array}{l} {\text{Km}}_{\text{AKG}}=2E-07{\text{x}}^{6}-2E-05{\text{x}}^{5}+0.000{\text{x}}^{4}-0.012{\text{x}}^{3}\\ \;+0.126{\text{x}}^{2}-0.634\text{x}+1.277 \end{array}$$

The coefficient of determination for these curves approximated unity (R^2^_Fe_ = 0.981, R^2^_AKG_ = 0.964), whilst the variance (σ^2^_Fe_ = 0.070, σ^2^_AKG_ = 0.025) of the raw datasets indicate a marginally greater dispersion of the iron affinity data. These parameters were used to select a particular model.

### Model evaluation

The models are robust (Figure 2C, Tables T2, 3A in Supplementary Material) with MM constants, *Km_Fe_* ≅ 0.644 ± 1.11*E* − 05 *mM* and *Km_AKG_* ≅ 0.784 ± 1.47*E* − 05 *mM*, when tested with previously defined ferrous iron concentrations. The performance of the model (NLR-Km/Ka_(Fe(II))) was examined with reference to the chi-squared values in accordance with Equation (1). The data was not significant for lower cytosolic levels of Fe (II), ({χ^2^_0.0005_, χ^2^_0.001_, χ^2^_0.0035_} > χ^2^_*crit*_; *p* > 0.05; *df* = 16) (Table T3B in Supplementary Material), implying a good correspondence between the NLR for *Km_Fe_* and kinetic data for various 2-oxoglutarate-dependent enzymes at these concentrations.

### Derived kinetic data

Two sets of data points were computed when used in association with Equation (2) and at previously defined threshold (*t*) values: (a) The distribution of extrapolated reaction velocities of enzymes (Figure 2D, Table T3B in Supplementary Material) and the model (Table 3C in Supplementary Material), and (b) Chi-squared (χ^2^) values comparing the extra- and intra-polated data (observed vs. model) (Tables T3D,E in Supplementary Material).

### Catalytic domain prediction

**Figure 3:**
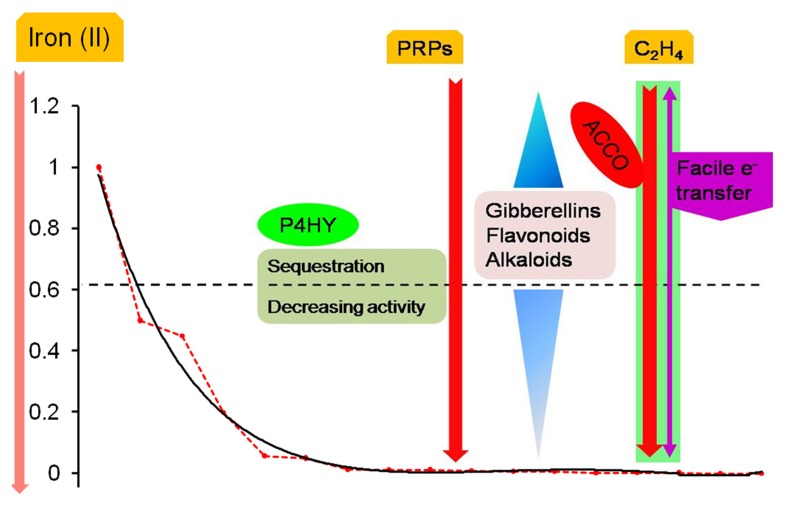
**Biochemical machinery during compensated/ early onset iron deficiency**. As the cytosolic levels of ferrous iron decline, the differential activity profile of 2OG-dependent enzymes influences the cellular response. These are depicted in reference to the non-linear regression model of predicted iron affinity data (*NLR_Km_Fe_*). Key players include prolyl 4-hydroxylase, despite being almost entirely organellar; secondary metabolites (GAs, bioflavonoids, alkaloids) and monotonic incremental- and decremental-responders; and dual-origin ethylene formation (enzymatic and FETs). Much of this cellular biochemistry is directed toward diminishing the resistance of the cell wall in preparation for growth, development, and the release of rhizosphere influencing metabolites.

**Figure 4:**
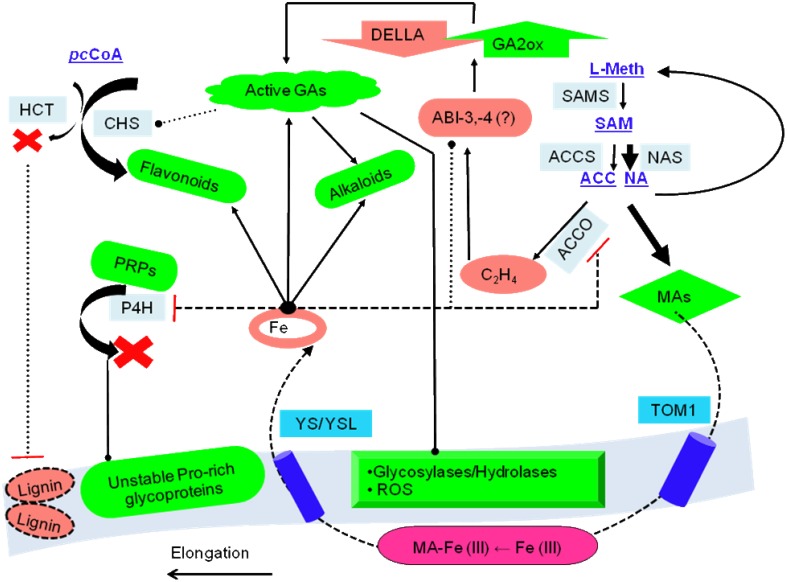
**Consolidated model of 2-oxoglutarate sensing and actuation in root hairs of graminaceous plants**. The role of intermediate metabolites of two major metabolic pathways: SAM to either ACC or NA, and p-coumaroyl-CoA(pCC) to either naringenin (flavonoids/isoflavonoids) or lignins, is presented. These ideas are centered on the non-catalytic electron transfers that characterize ACCO, sensor binding and information transfer by transcriptional factors, and kinetic data (existent and predicted). The model predicates root hair elongation as a function of cell wall loosening with concomitant iron -chelation and –pool restoration. Key: regulation (….), low activity (----). Abbreviations: PRPs, proline-rich glucoproteins; TOM1, transporter of the major facilitator superfamily; YS/YSL, yellow-stripe/ yellow-stripe like; ROS, reactive oxygen species; L-meth, L-methionine; pDELL, proteins with the N-terminal DELLA domain (RGA, RGL1, GAI, RGL2, RGL3); ABI, Absicisic acid insensitive; CHS, chalcone synthase; HCT, hydroxycinnamoyl-CoA: shikimate/quinate hyroxycinnamoyl-CoA transferase; pcCoA, p-coumaroyl-CoA; MAs, mugineic acids.

**Figure 5:**
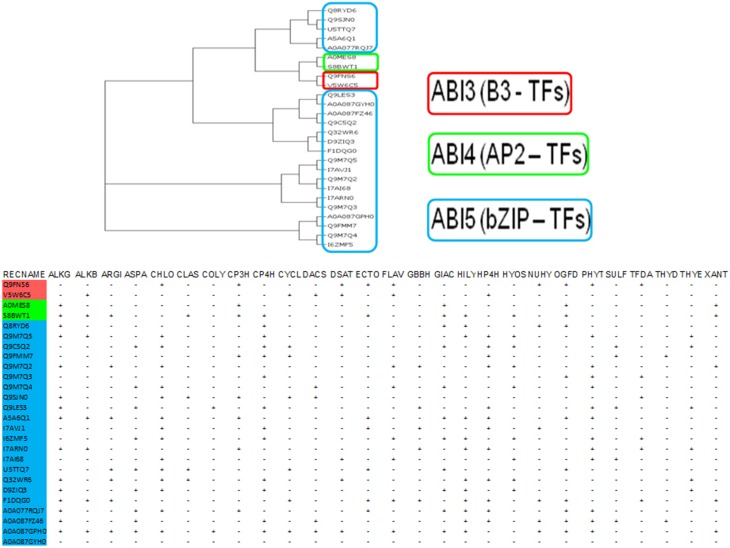
**Analysis of the protein products of Absicisic-acid insensitive (ABIs −3, −4, and −5) mutant causing genes**. These function as integrators of stimulus-responsive cascades. This is in part, due to a bimodal binding pattern, i.e., cytosolic elements and DNA-binding, that typifies many of these proteins. Evidence indicates that the non-catalytic ABIs form intra- and inter- protein networks, and regulate their cytosolic-nuclear levels by forming self-limiting feedback mechanisms. Domain analysis of these sequences for 2-OG binding motifs suggests the presence of basic (DNA-binding, B3; AP2; bZIP) and acidic amino acids which may coordinate iron and/or form protein-protein interactions with well characterized 2-oxoglutarate members. (#) Abbreviations: Kundu, 2012: ALKB, Alk-B like demethylase; ARGI, Arginine hydroxylase; ASPA, Aspartyl:Asparaginyl hydroxylase; CHLO, Chlorination; CLAS, Clavaminate synthase; COLY, Collagen lysyl dioxygenase; CP3H, Collagen prolyl 3-hydroxylase; CP4H, Collagen prolyl 4-hydroxylase; CYCL, Cyclization; DACS, Deacetoxycephalosporin-C synthase; DSAT, Desaturase; ECTO, Ectoine hydroxylase; FLAV, 2S-Flavones; GBBH, *γ* – butyrobetaine hydroxylase; GIAC, Gibberellic acid; HILY, Histone lysyl demethylase; HP4H, Hypoxia prolyl 4-hydroxylase; HYOS, Hyoscyamine; NUHY, Nucleotide/side hydroxylase; OGFD, Eukaryotic initiation factor 2α (eIF2α); PHYT, Phytanoyl-CoA; SULF, Sulfate cleaving; TFDA, 2,4-Diphenoxyacetic acid metabolizing; THYD, Thymidine dioxygenase; THYE, Thymine dioxygenase; XANT, Xanthine hydroxylase.

A biological response when graded temporally might be categorized as early (minutes to hours) or late (days), and is characterized by sequential changes in the levels of stored and newly synthesized proteins (Figures 3, 4). An analysis of putative protein products of the transcription factor genes (ABI −3, −4, −5), implicated in the development of absicisic acid (ABA) insensitive mutants (*N* = 26; Table T4A; S1A, S1B, S2 in Supplementary Material) was undertaken to map 2OG function onto these sequences. An examination suggests that the generic AKG domain is present in a majority of sequences (≈ 57%, *N* = 15). Interestingly, only putative ABI4- (100%, *N* = 2) and ABI5- (≈ 60%, *N* = 13) sequences possess the same (Figure 5). A distribution of substrate specific specialized regions is tabulated (Table T4B in Supplementary Material; Figure 5).

## Discussion

Compensated iron deficiency is an orchestrated set of molecular steps that cells undertake to reserve essential elements for the most critical of functions. This cellular triage, at least in the roots of grasses, could be the result of initialization-, consolidation-, and termination-level intervention by the AKG-dependent dioxygenases. Any model would have to be consistent (coefficients are treated as constraints), yet varied (an error term and/or a stochastic event), and function with both intra- and inter-systemic components.

### Model description and function

NLR-models are specific for a particular system, accurate, and limited to a single independent variable. Levels of iron in external media have been used previously as a method of classifying the effects on the host cell (iron-saturating, 1μ*M* < *t* ≤ 20 μ*M*; iron-deficiency, 0.5 μ*M* < *t* ≤ 1μ*M*; iron-limited, *t* ≤ 0.5 μ*M*; Urzica et al., 2012). These concentrations were utilized as reference values for the NLR-models in this study. (Table T2 in Supplementary Material). The labile iron pool, despite its *in vivo* relevance is relatively inestimable, given the large number of macromolecules and pathways that utilize iron, and was therefore, not considered. There is a thin line between a continually depreciating iron level, as a physiological stimulus to compensatory mechanisms and, as an inducer of stress response pathways. Whilst, the former, entails a metabolic re-distribution mediated by real-time selection (a deficiency state), the latter results in observable structural alterations (a limited state). Although, there is biochemical proof of this delineation (Schikora and Schmidt, 2001a; Urzica et al., 2012), an analytic treatment of this phenomena could provide valuable insights into the nature of this dynamic molecular interplay.

Here, I have constructed statistical non-linear regression models of existing biochemical data (Figures 2A,B, Table T2 in Supplementary Material) for both iron (II) and 2-oxoglutarate (NLR-Km/Ka_(Fe(II)); NLR-(Km/Ka_2OG), and proceeded, via inference, to unravel the underlying molecular complexity. The models were evaluated for goodness of fit, measure of dispersion, and robustness. The conserved jelly-roll fold that characterizes this superfamily presents active site residues that interact with Fe(II) and alpha-ketoglutarate (Figures 1A,B). However, selected data from mutagenesis experiments indicates that, while iron co-ordination is central to catalytic activity, the demand for 2OG as a co-substrate is less stringent (Brisson et al., 2012). Many enzymes, have an ancillary need for ascorbate to preserve the active site occupancy of iron (II) (Fe^3+^ to Fe^2+^). The rationale for selecting the NLR_Km/Ka_(Fe(II)) model as a suitable representation was: a) stringency, symbolized by a map equating the coefficient of determination (*R*^2^) of the chosen NLR-models and the datasets to the frequency of iron or 2OG binding by the common fold, i.e.,

(5) $$R^{2}\left(D_{z}\right)\mapsto \;\phi_{z}$$

ϕ = necessity of enzyme modifier to the reaction

z ∈ {Fe, AKG}

Here, ϕ_*Fe*_ = 1 and ϕ_*AKG*_ <1 (by definition); and b) presence of dispersed data. Since the objective was a model which factors in the unequivocal presence of the co-factor or—substrate and accounts for the broad range of Km values (proportionate to the number of postulated molecular roles), these parameters are perfectly credible as screening metrics. Computations (R^2^_Fe_ > R^2^_AKG_; σ^2^_Fe_ > σ^2^_AKG_), indicate that the affinity of 2OG-dependent enzymes for iron could influence their propensity to work synergistically (Kundu, 2015).

### The 2OG-dependent superfamily as an interactome

The dominant character of this transient interaction could have important consequences for cell physiology (Kundu, 2015). The conserved nature of the amino acids of the greek-key motif/ jelly- roll fold that are responsible for coordinating iron, intuitively suggests a tight regulatory mechanism(s). Consequently, iron (II) could function as a critical switch in the genesis of a suitable adaptive response, either involving AKG-dependent enzymes exclusively, or in-tandem with downstream mediators (nuclear-cytoplasmic receptors, transcription factors; Bai et al., 2012).

#### Rapid and early response to ebbing iron levels

##### Reduced affinity for iron as a suitable sensor

2OG-dependent catalysis is characterized by an absolute need for Fe(II), as opposed to a facultative role for AKG. Despite this, it was surprising to note that the affinity spectrum for iron spans three orders of magnitude (log*^Km_max_^*/*_Km_min__* ≅ 3.301; Table T1A in Supplementary Material). Given the sequence and structural variants thereof, this was not entirely unexpected. This finding, however, implies a lack of robustness, or reciprocally, a heightened sensitivity to fluctuations in cytosolic iron among low affinity (Class II), members of this superfamily. A comparison between the Michaelis-Menten (MM) constant for the model (NLR-Km/Ka_Fe(II)) and GA2ox (*Km_max_*), 0.644 vs. 1.00 mM, indicates that the reduced activity of this enzyme may be the earliest effected modality of the root hair cell in its reaction to iron deprivation. Since GA2ox is responsible for the catabolism of the bioactive gibberellic acids, the below-par activity (*V*^0^_*G*2OX_ (0.644) ≅ (0.3) *V_max_*; *V*^0^_*G*3OX_ (0.644) ≅ (1.6) *V_max_*) (Table T1 in Supplementary Material, Figure 2D), would result in the accumulation of GAs. This primary- and secondary- (increased ubiquitinylation of DELLA-domain proteins; early-gene hypothesis; Zentella et al., 2007) inflation of GA levels could result in a bias favoring flavonoid biosynthesis in competing substrate pathways (Figures 3, 4), and alkaloid metabolism.

##### Priming the cell for compensatory redirection

The phase of iron deficiency (0.0005–0.001 mM) is characterized by a low predicted *Km* value for the model (*V*^0^_*NLR_Km(Fe)*_ (0.001) ≤ (0.0008)*V_max_*) and is as remarkable for its biochemical re-routing, as is its inertness to any major cytoskeletal alterations. The predicted enzyme velocity values however, suggest that with the exceptions of asparagine hydroxylase (*V*^0^_*ASHY*_ (0.001) = *V_max_*), and thymine dioxygenase (*V*^0^_*THYE*_ (0.001) ≅ *V_max_*/2), the catalytic transformation is sub-optimal (*V*^0^_*max*_ < *V_max_*/2) for every other Class I member. Interestingly, both these enzymes are important mediators of nuclear-signal transduction. While, ASHY fulfills this role in association with P4HY to impel a cellular response to hypoxia, THYE does so indirectly by catalyzing the oxygen-sensitive committed first reaction in the synthesis of the modified base J (Yu et al., 2007; Vainio et al., 2009). Significant though these changes maybe, the shuffling of metabolic pathways to preserve critical function is probably hormonal in origin (cascadic, non-local, low specificity, and self-limiting).

A major convergent node to emerge during this phase is the loss of integrity of the cell wall. This cytoskeletal entity, despite its *de facto* physiological role in transport is remarkably predisposed to biochemical maneuvering. Three major classes of 2OG-dependent molecules in graminaceous roots are likely to effect the utilization of iron by switching between sub- (reversible) and supra- (committed) threshold states. The cumulative actions of the elevated bioactive GAs (Hedden and Phillips, 2000a,b), continued synthesis of ethylene, and the sequestered prolyl 4-hydroxylase, result in the activation of several anti-parallel reaction pathways. Cell wall loosening is probably implemented directly by GA- and ethylene-activated resident hydrolases or expansion proteins (Carpita and Kanabus, 1988; Cho and Cosgrove, 2002; Cui et al., 2005), or by a deficient PRP yield (Hijazi et al., 2014), and indirectly, consequent to a flux in the direction of the bioflavonoid synthetic pathway (Figures 3, 4). These pro-enervating signals are kept in check by the potent antioxidant activity of the polyhydroxylated 2S-flavonols (Kumar and Pandey, 2013).

Ethylene, a powerful gas acts as dominant negative molecular regulator of senescence and ripening. At the observed iron (II) concentrations for compensation (<0.001 mM), the major ethylene synthesizing enzyme,1-amino-cyclopropane-carboxylate-oxidase (ACCO, EC1.14.11.4), has almost null activity (*V*^0^_*ACCO*_ (0.001) < (0.008) *V_max_*). Despite this, facile electron transfers (FETs) aided by the relatively more compact ACC's continued presence at the active site (Zhang et al., 2004) and its proximity to O_2_, results in an almost unabated egress of ethylene (≈ 0.35 mol; Rocklin et al., 2004; Mirica and Klinman, 2008). This non-enzymatic transformation also results in activated molecular dioxygen diffusing out of the active site, and a progressive increase in flux of the precursor SAM toward the synthesis of the mugineic acids (Ma et al., 1995) (Figure 3).

Yet another objective accomplished by these pathway rearrangements is the provision of a readily accessible nitrogen source. Bioflavonoids and alkaloids are important secondary metabolites synthesized in plants (Zhang et al., 2012; Long et al., 2013). 2OG-dependent enzymes are known to impact the tropane, isoquinoline, and monoterpenoid-indole classes of alkaloids (Table T1A in Supplementary Material) (Matsuda et al., 1991; Vazquez-Flota et al., 1997; Hagel and Facchini, 2010a,b). Predicted affinity data for individual enzyme systems (*V*^0^_*FLVS*_≅ (56) *V_max_*; *V*^0^_*VINS*_≅ (74)*V_max_*,) demonstrate that at these levels of ferrous iron, secondary metabolite synthesis is still functioning above par (Figures 3, 4).

##### Structural changes in uncompensated iron deficiency

Empirical data (Urzica et al., 2012) suggests that as the exogenous and thereby, cytosolic iron falls to less than 0.0005 mM, there is a total and irreversible loss of cell wall integrity. The molecular actuator(s) of this watershed event are debatable, with an imbalance, rather than the perturbation of any isolated analyte, being causal.

The gibberellic acid (GA) mediated flux and consumption of p-Coumaroyl-CoA (pCC) in favor of 2S-flavanol synthesis at higher cytosolic ferrous iron levels constitutes a fundamental branch point. Whilst, the compromised monolignol levels may accelerate cell wall weakening, the routing of pCC toward other pathways, notably coumarin synthesis via feruloyl-CoA, would equip the cell with another means to chelate Fe(III) in the presence of a high pH soil (Kai et al., 2008; Schmid et al., 2014). However, since, these reactions are Fe(II)-dependent, the continually dipping levels of this essential trace element could result in the near-complete cessation of activity of 2S-flavanol synthetic enzymes (*V*^0^_*FLVS*_ (0.001) ≅ (0.05)*V_max_*; *V*^0^_*FLVS*_ (0.0005) ≅ (0.02)*V_max_*). The physical presence of ferrous iron at the active site of high affinity 2OG-dependent catalysts might also be expected to contribute to the formation of free radicals in a manner reminiscent of ACCO activity. FETs in many of these enzymes could convert O_2_ into potent sources of ROS/RNS (Muller et al., 2009). Reactive –oxygen and –nitrogen species are a group of related free radicals, *vide*., hydroxyl- (OH^−^), superoxide- (O^2−.^), nitrite- (NOO^−^), and peroxynitrite- (ONOO^−^) anions. The predicted velocity maxima, (*V*^0^_*NLR_Km(Fe)*_ (0.0005) ≅ (0.0004)*V_max_*), hints at the possibility that such an episode may indeed transpire.

This unchecked increase in the concentration of ROS/RNS consequent to (i) the diminishing pool of free-radical scavenging bioflavonoids, and (ii) residual enzyme FETs, could constitute the final insult to the integrity of the cell wall propelling it toward a state of null resistance, thereby, (a) expediting the growth and elongation of immature zonal cells with the formation of root hairs, (b) nourishing newly formed tissues by depleting the alkaloid reserves, and (c) expelling exudates enriched in the mugineic acids (2′-DMA, MA, 3′-HMA, 3′-hydroxymugineic acid; 3′-EMA, 3′-epihydroxymugineic acid) into the surrounding rhizosphere. These can, then solubilize the ferric iron and effect an inward transport mediated by the proteins YS/YSL (yellow stripe/yellow stripe like) restoring cytosolic iron (Nakanishi et al., 2000; DiDonato et al., 2004; Koike et al., 2004; Nozoye et al., 2011) (Figure 4).

#### DNA binding proteins as long term signal transducers of iron deprivation

An emerging line of evidence suggests under conditions of abiotic stress such as cold, drought, and/or excessive salinity, the AP2 family of transcriptional regulators function to mitigate the effects of the noxious stimulus by slowing growth and preserving resources (Achard et al., 2008; Magome et al., 2008; Zawaski and Busov, 2014). Absicisic acid (ABA) insensitive mutants, have variations in defined loci and code for phosphatases (ABIs-1, −2), transcription factors (ABIs −3, −4, −5), and proteins with miscellaneous function (ERA -1, -3), that allow them to thrive at concentrations that favor seed dormancy (Koornneef et al., 1984; Cutler et al., 1996; Leung et al., 1997; Finkelstein et al., 1998; Alonso et al., 1999; Brady et al., 2003). ABI3 (IDEF2, iron deficiency 2), a B3 containing modulator may bind to divalent cations (Fe^2+^, Cu^2+^, Ni^2+^) directly, and sense the proportion of iron. This is translated into a differential signal for downstream gene regulation (Kobayashi et al., 2007, 2010, 2012).

In this work although the sample size was small, none of the putative ABI3 sequences possess the AKG catalytic region. ABI4, a member of the AP2-family of transcriptions factors (TFs), may function by (a) binding to and upregulating a GA2ox, and/or (b) proteasomal degradation of key DELLA proteins (Finkelstein et al., 2011; Cantoro et al., 2013). ABI5, a basic zipper transcription factor (bZIP), has a conserved alanine residue which interacts with ABI3. ABI5 mutants exhibit increased tolerance to osmotic stress (NaCl and mannitol; Ogo et al., 2007; Tezuka et al., 2013). Since, a majority of ABI5 sequences also contain the AKG domain, it is reasonable to infer that the concentration of ABI5 is governed by co-factor dependence, which in turn may effect formation/stability of the ABI3-ABI5 complex, and the transactivation of the IDE response genes, thereof.

#### Concatenating the temporally distinct pathways

Whilst, the 2OG-dependent response has its fulcrate on the presence of pre-formed molecules, and is in most cases self-limiting, there is little clarity on the contribution of TFs in a unified model. One possibility could be the tight binding of an AKG-dependent member to a TF. As levels of ferrous iron plummet, this interaction could become less restrictive due to allosteric mechanisms. In such as scenario, the TF could then enter the nucleus and execute the upregulation of other genes (Itai et al., 2013). Another attractive hypothesis could be that some of these TFs (ABIs −3, −4, and/or −5) may possess latent 2OG-dependent activity and/or, may present a favorable protein-protein interaction surface with other 2OG-dependent enzymes. An AKG-domain analysis of putative 2OG-dependent ABIs (Figure 5; Table T4B in Supplementary Material), reveals that the histone demethylase (HILY), hypoxia prolyl 4-hydroxylase (HP4H), and the 2-oxoglutarate and iron-dependent dioxygenase domain containing 1 (OGFOD1) domains are among the most frequent (> *q*3). These domains, as components of characterized enzymes are well established in literature as mediators of cytosolic-nuclear factor shuttling that terminates in transcriptional regulation of stress inducible genes (Myllyharju, 2008; Wehner et al., 2010; Thinnes et al., 2014).

**Figure 6:**
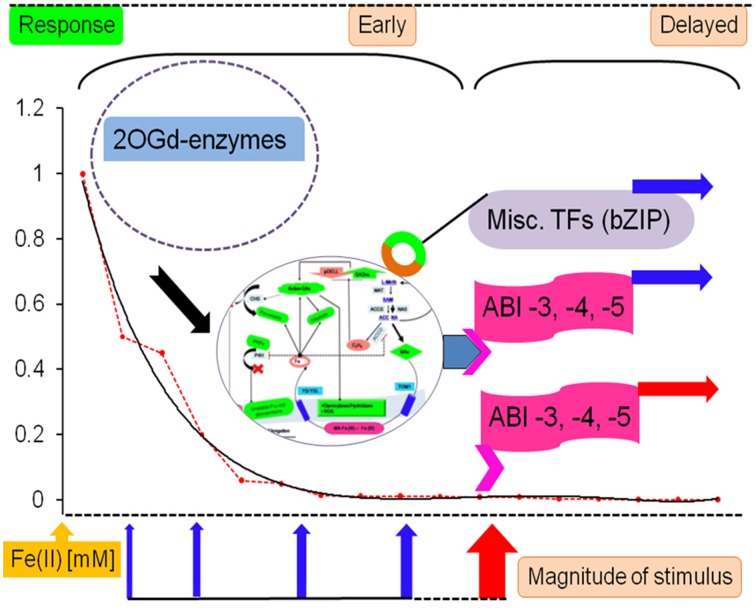
**AKG-dependent enzymes as a molecular glue in the response to iron deficiency**. A key threshold value for cytosolic ferrous iron, 0.0005 mM, governs the response route of the root cell. Stimuli leading to steady-state levels above this, elicit attenuated counter-measures, that are limited to metabolic redirection. However, below this value structural alterations are common. These long-term changes are mediated by the transcription factors (ABI −3, −4, −5) and can be influenced both, by an instant deprivation of great proportion or an uncompensated threshold cross-over. TFs are also thought to be evoked in previous stages by the phytohormones (Schikora and Schmidt, 2001b). Abbreviations: 2OGd, 2-oxoglutarate-dependent; TFs, transcription factors; Fe (II), ferrous iron; ABI, absicisic acid insensitive.

From the above data it does seem conceivable that TFs could indeed serve as a critical component of a systems level 2OG-dependent reaction to iron-deficiency, both as a sensor and actuator of a late iron-deficiency response (Kobayashi et al., 2012). This advanced involvement of the transcriptional apparatus, directly (IDEF2) and/or indirectly (ABI4, ABI5), would have to be synchronized with the early loss of GA2ox activity, secondary metabolite accumulation, and compromised cell wall integrity. This could ensure new and continued elongation of root hairs, whilst transporting Fe (III)-MA chelates internally (Figure 6).

## Conclusions

Previous work has purported the origin of a stimulus-driven dynamic network of 2-oxoglutarate dependent enzymes to accomplish system-level function, a novel role for this superfamily. The multitude of substrates and reactions that characterize these catalysts were deemed responsible for this expression. The models and analysis presented, *vide supra*, provide proof-of-concept of the assertion that a combinatorial assembly of 2OG-dependent enzymes can manifest within the cellular milieu and negate the effects of an external perturbation, such as the deprivation of an important micronutrient. The co-operative intermolecular kinetics of this association results in directed quasi-pathway(s) with: feedback regulation, compartmentalization, and product utilization, as component modifiers. Although the model(s) are sufficiently coarse-grained, details of the underlying stoichiometry (product(s), reactant(s)) remain elusive. The measure of flux, of these metabolites, too, is not elaborated on. Further analysis to elucidate these details could be undertaken, as could be the deciphering of the kinetic constants from available atomic-scale data.

## Author contributions

SK collated all data, carried out the computational analysis, formulated and refined the models, wrote all relevant code, and the manuscript.

### Conflict of interest statement

The work presented here has not been funded by any agency, has been done during the personal time of the author, and with the author's own resources. The author declares that the research was conducted in the absence of any commercial or financial relationships that could be construed as a potential conflict of interest.

## Supplementary material

Table T1 **Kinetic data for Fe(II) and 2-oxoglutarate (T1.xlsx)**.

Table T2 **Datasets of computational experiments using the Monte Carlo protocol (T2.xlsx)**.

Table T3 **Robustness of NLR models across predefined thresholds of cytosolic iron (T3.xlsx)**.

Table T4 **Distribution of catalytic domains in select sequence homologs of proteins responsible for ABA insensitivity (T4.xlsx)**.

S1A, S1B **Computed HMMER details of domains in Table T4 (^*^.zip, ^*^.txt)**.

S2 **ABI-3, −4, and −5 sequences in fasta format (^*^.txt)**.

## Abbreviations

2OG: 2-oxoglutarate
AKG: α-ketoglutarate
2OGd: 2OG-dependent
*Km*: Michaelis-Menten constant
ROS: Reactive oxygen species
RNS: Reactive nitrogen species
MAs: Mugineic acids
ABI: Absicisic acid insensitive.
